# Improving peak concentrations of a single dose regime of gentamicin in patients with sepsis in the emergency department

**DOI:** 10.1371/journal.pone.0210012

**Published:** 2019-01-22

**Authors:** Maarten Cobussen, Patricia M. Stassen, Dirk Posthouwer, Frank H. van Tiel, Paul H. M. Savelkoul, Thomas Havenith, Michiel B. Haeseker

**Affiliations:** 1 Department of Medical Microbiology, Maastricht University Medical Centre, Maastricht, the Netherlands; 2 Department of Internal Medicine, Maastricht University Medical Centre, Maastricht, the Netherlands; 3 Department of Clinical Pharmacy, Maastricht University Medical Centre, Maastricht, the Netherlands; University of Nottingham School of Medicine, UNITED KINGDOM

## Abstract

**Objective:**

To achieve an optimal effect in patients with sepsis at the emergency department (ED), the gentamicin peak-concentration should be sufficiently high (i.e. peak-concentration/MIC ≥8–10). ICU patients with sepsis often need higher gentamicin doses to achieve sufficiently high peak-concentrations. The aim of this study is to investigate which dose is needed to reach adequate peak-concentrations in patients presenting with sepsis at the ED.

**Methods:**

Patients with sepsis at the ED were included from August 2015 until February 2017. Peak-concentrations were measured in blood 30 minutes after the first gentamicin dose. The study consisted of three phases. In the first phase, peak-concentrations were measured after a standard dose of 5mg/kg. In the second phase, a simulation ((peak-concentration/actual dose) × simulated dose) was performed to determine which dose was needed to reach adequate gentamicin peak-concentrations of ≥16mg/L. In the third phase, peak-concentrations were measured for the best simulated dose.

**Results:**

In phase one, of 86 patients who received a dose of 5mg/kg, 34 (39.5%) patients did not reach the target peak-concentration of ≥16mg/L, and 73 (84.9%) did not reach ≥20mg/L. In phase two, the simulation showed that with a dose of 7mg/kg 83 (96.5%) patients would reach peak-concentrations ≥16mg/L, and 67 (77.9%) of ≥20mg/L. In phase three, 53 patients received a dose of 7mg/kg, of whom 45 (84.9%) reached peak-concentrations of ≥16mg/L, and 31 (58.5%) of ≥20mg/L.

**Conclusion:**

Patients with sepsis at the ED need higher doses of gentamicin. A dose of 7mg/kg is needed to achieve adequate peak-concentrations in the majority of patients.

## Introduction

Sepsis is associated with high morbidity and mortality [1]. Aminoglycosides, such as gentamicin, are frequently used as empirical treatment of sepsis at the emergency department (ED). Generally, aminoglycosides are combined with broad spectrum β-lactam antibiotics to broaden the Gram negative spectrum. Early administration of empirical antibiotic therapy within one hour of presentation in patients with sepsis is essential to improve outcome [2].

Gentamicin is a bactericidal antibiotic with concentration dependent pharmacodynamic properties. In order to achieve a maximal bactericidal effect of gentamicin, it is recommended that the ratio between the peak concentration and the minimal inhibitory concentration (MIC) must be 8–10×MIC [3, 4]. The height of the peak concentration corresponds with the clinical response rate and is approximately 85% with a ratio of 8:1, and 90% with a ratio of 10:1 [5, 6]. According to the European Committee on Antimicrobial Susceptibility Testing (EUCAST), gentamicin MIC breakpoints for Enterobacteriaceae are ≤2mg/L, which leads to a target peak concentration of 16-20mg/L for gentamicin susceptible isolates [7].

Critically ill patients at the intensive care unit (ICU) do not reach an adequate peak concentration of ≥16mg/L after a gentamicin dose of 5mg/kg [8, 9], which is most probably due to a larger volume of distribution (Vd) of these patients compared to healthy subjects [8, 10–13]. This larger Vd leads to lower gentamicin peak concentrations, and therefore a higher dose of ≥7mg/kg has been advised in these patients [9]. To our knowledge, all studies have been performed in ICU patients and so far, no studies have been performed in patients with sepsis at the ED. However, it is likely that patients with sepsis at the ED also have a larger Vd and also need a higher dose of gentamicin (e.g. >5mg/kg).

To investigate the gentamicin dose that leads to an adequate peak concentration in patients with sepsis at the ED, we measured gentamicin peak concentrations in patients with sepsis at the ED after the first dose of 5mg/kg gentamicin to determine whether target peak concentrations were reached in these patients. We further determined which dose was necessary to achieve target peak concentrations in a simulated model and subsequently tested this simulated dose to validate the simulated dose.

## Material and methods

### Study population

We performed a prospective study in the Maastricht University Medical Centre (MUMC). Patients ≥18 years of age presenting with sepsis, according to the SIRS criteria for sepsis [14], at the ED from August 2015 to February 2017, that were treated with broad spectrum β-lactam antibiotics (usually amoxicillin/clavulanic acid 1000/200mg q6h) and gentamicin intravenously (5 or 7mg/kg once daily) [15], with a peak serum concentration available after the first dose, were included. For patients with a BMI >30kg/m^2^, the adjusted body weight was used to calculate the dose of gentamicin [16]. Patients were excluded when serum peak concentrations were not obtained according to protocol.

In our hospital, a practical approach is to give a single dose of an aminoglycoside in combination with broad spectrum β-lactam antibiotics to patients presenting with severe sepsis or septic shock in the ED, to re-evaluate after 24 hours and then decide whether or not to repeat the aminoglycoside dosing, based on the clinical condition of the patient and the follow-up laboratory results. A substantial number of hospitals in the Netherlands, including ours, use this strategy.

During the study period the sepsis criteria were renewed from the SIRS criteria to the SOFA criteria and we retrieved data for both criteria [17]. For inclusion, patients would have to meet the SIRS criteria for sepsis.

### Data collection

All data were retrieved from the electronic hospital charts and the hospital’s microbiological database. Standardised scoring forms were used to extract the data from the charts. We recorded age, sex, weight, length, vital parameters, gentamicin dose, date and time of gentamicin peak concentration, and microbiological data. In addition to the SIRS criteria for sepsis [14], the new Sepsis-3 criteria, the SOFA criteria, were retrieved [17].

### Pharmacokinetic/pharmacodynamic analysis

Our study was performed in three consecutive phases. In phase one, gentamicin peak concentrations were measured in patients with sepsis at the ED who had received the standard gentamicin dose of 5mg/kg. Gentamicin was infused over 30 minutes. The gentamicin blood concentrations were drawn 30 minutes after the gentamicin infusion was stopped. According to the EUCAST the gentamicin clinical breakpoint for Enterobacteriaceae is 2 mg/L. This means that micro-organisms with an MIC ≤2mg/L are susceptible for gentamicin. The PD target peak concentration should be at 8 × 2 mg/L = 16 mg/L, or even better 10 × 2 mg/L = 20 mg/L. Therefore, a gentamicin peak concentration of ≥16mg/L was defined as adequate [3, 4]. The Vd was calculated with the formula: ‘Vd (L/kg) = gentamicin peak concentration (mg/L) / gentamicin dose (mg/kg)’.

A validated fluorescence polarization immunoassay (Roche Cobas Integra 800) was used to measure the gentamicin peak concentrations. The lower threshold of detection was 0.2mg/L, the upper threshold of detection was 9.0mg/L, and at 0.3mg/L, the coefficient of variation was 8.5%. At 1, 4, and 8mg/L, the coefficient of variation was 0.9%, 1.7% and 2.9%, respectively. Samples of peak concentrations were manually diluted before analysis.

In phase two, peak concentrations were simulated for different dosage regimens using the Vd and peak concentrations of the patients in phase one. Simulations for doses of 6 to 10 mg/kg (“simulated dose”) were performed to calculate the “peak concentration simulated dose” with the formula: ‘peak concentration simulated dose = (peak concentration/actual dose) × simulated dose’.

In phase three, a new gentamicin dosing regimen with the best simulated dose was implemented in our hospital to confirm the simulations of that dose. The best simulated dose was defined as that dose with which >95% would reach a peak concentration of >16 mg/L. After implementation, gentamicin peak concentrations were measured in a new group of patients with sepsis receiving the best simulated gentamicin dose, to confirm whether the desired target peak concentrations were reached.

### Renal function

Although our study was not designed to assess the risk of nephrotoxicity, we investigated whether increasing the dose of gentamicin from the standard dose to the best simulated dose would lead to an increase in nephrotoxic side effects, and a higher occurrence of acute kidney injury (AKI). Serum creatinine values were retrieved at the moment of presentation at the ED and followed for up to 14 days when available. To assess baseline renal function, creatinine values up to 3 months prior to ED presentation were retrieved. When these creatinine values were not available, we used the lowest creatinine value during admission as baseline creatinine [18]. AKI was classified according to the Risk, Injury, Failure, Loss of function and End stage renal disease (RIFLE) criteria using serum creatinine values [19]. The occurrence of AKI was evaluated on three different time periods (T1: 1–2 days after admission, T2: 3–7 days after admission, T3: 8–14 days after admission) during 14 days of follow up. AKI on admission was defined as a decrease in kidney function at the ED relative to baseline creatinine value. When patients received concomitant renal replacement therapy or had a post-renal cause of AKI they were excluded from analysis of the occurrence of AKI.

### Microbiological analysis

All positive blood cultures from ED patients in 2015 with Gram negative bacteria were retrieved for the analysis of MIC distributions. Identification of bacteria in the blood cultures was performed using Becton Dickinson Phoenix Automated Microbiology System (USA). The MIC was measured with the Phoenix or E-test (bioMerieux, France).

### Statistical analysis

Statistical analysis was made using IBM SPSS version 22 (SPSS Inc., USA). Continuous variables were reported as median [interquartile range (IQR)], and categorical variables as proportions. For AKI, valid percentages were reported, since some creatinine values were missing during follow up. Comparisons between two groups were made using Mann-Whitney test for continuous non-Gaussian data and Pearson’s chi-squared test for categorical data. Linear regression analysis was used to test for correlation between simulated and true measured values. *P* values <0.05 were considered statistically significant.

### Ethical approval

The Ethics Committee of the Maastricht University Medical Centre reviewed and approved the protocol for this study and waived the need for consent. (METC 14-4-099).

## Results

### Study population

During phase 1, a total of 86 patients with sepsis receiving gentamicin in a dose of 5mg/kg was included. The median age was 66 [60–77] and 59.3% was male (Table 1). Median body weight was 76 kg [64–90] and median BMI was 25.3kg/mg^2^ [21.7–29.3]. All patients had sepsis according to the SIRS criteria. Severe sepsis was found in 25.6% of patients, and 10.5% had septic shock. According to the SOFA criteria, 46.5% of patients had sepsis, and 7.0% had septic shock.

**Table 1:** Characteristics of patients with a dosing of 5mg/kg and 7mg/kg.

n (%) or median [IQR]	5 mg/kg (phase 1) n = 86	7 mg/kg (phase 3) n = 53	*P* value
Male sex	51 (59.3)	35 (66.0)	0.48
Age (years)	66 [60–77]	72 [60–80]	0.24
Length (m)	1.70 [1.64–1.78]	1.70 [1.65–1.79]	0.92
Body weight (kg)	76 [64–90]	78 [67–89]	0.39
BMI (kg/m^2^)	25.3 [21.7–29.3]	26.5 [23.3–30.8]	0.21
Gentamicin dose (mg/kg)	5.0 [4.6–5.1]	6.9 [6.3–7.1]	
Gentamicin peak concentration (mg/L)	16.6 [14.7–18.8]	21.8 [18.0–26.0]	<0.001
Peak concentration <16 mg/L	34 (39.5)	8 (15.1)	0.002
Peak concentration <20 mg/L	73 (84.9)	22 (41.5)	<0.001
Volume of distribution (L/kg)	0.29 [0.26–0.34]	0.31 [0.26–0.38]	0.23
Sepsis (SIRS)			
Sepsis	55 (64.0)	42 (79.2)	0.06
Severe sepsis	22 (25.6)	9 (17.0)	0.30
Septic shock	9 (10.5)	2 (3.8)	0.21
Sepsis (SOFA)			
No sepsis	40 (46.5)	25 (47.2)	1.0
Sepsis	40 (46.5)	26 (49.1)	0.86
Septic shock	6 (7.0)	2 (3.8)	0.71
Baseline creatinine (μmol/L)	84 [71–116]	84 [68–108]	0.39
Baseline urea (mmol/L)	5.7 [3.7–10.8]	5.9 [3.8–7.8]	0.62
Admission creatinine (μmol/L)	90 [76–155]	95 [67–117]	0.22
Admission urea (mmol/L)	7.4 [4.4–10.8]	5.8 [4.9–9.5]	0.63

BMI: body mass index; C_max_: peak concentration; SIRS: systemic inflammatory response syndrome [14]; SOFA: sequential organ failure assessment [17].

Median baseline creatinine prior to admission was 84μmol/L [71–116]. On admission, serum creatinine was 91μmol/L [77–158] (Table 1).

### Phase 1: Determining adequacy of peak concentrations with standard gentamicin dose 5mg/kg

**Fig 1:**
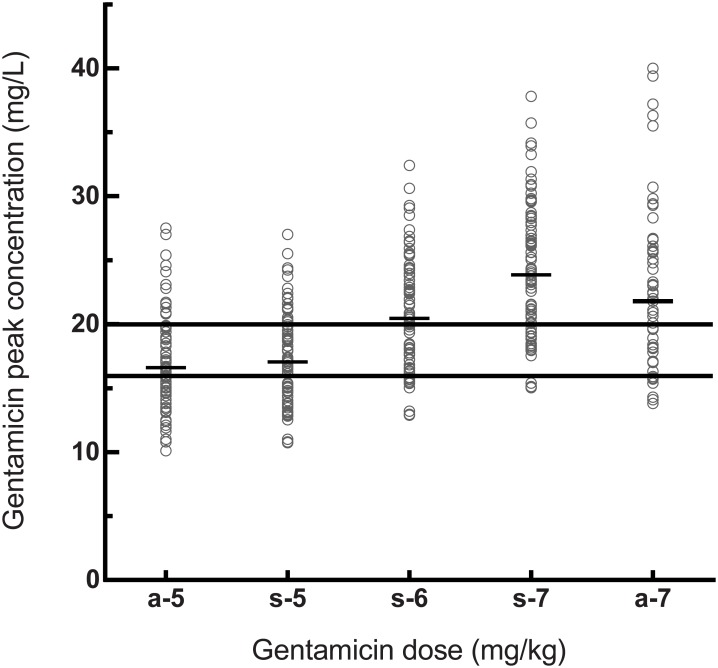
Gentamicin peak concentrations for different actual and simulated doses. a-5: actual given dose 5 mg/kg (phase 1); a-7: actual given dose 7 mg/kg (phase 3); s-5 –s-7: simulated doses for 5–7 mg/kg (phase 2). The short black line represents the median peak concentration in mg/L of every dose. The long black lines mark the target peak concentration of 16–20 mg/L.

Patients were given a median first dose of 5.0 mg/kg [4.6–5.1] with a Vd of 0.29 L/kg [0.26–0.34] (Table 1). A median gentamicin peak concentration of 16.6mg/L [14.7–18.8] was measured in these patients. With this gentamicin dosing regimen of 5mg/kg, 34 (39.5%) patients did not reach the advised peak concentration of at least 16mg/L, and 73 (84.9%) patients did not reach a peak concentration of >20mg/L (Table 2 and Fig 1).

**Table 2:** Actual (a-5 and a-7) and simulated (s5 –s10) gentamicin peak concentrations in n (%).

	a-5 mg/kg	s-5 mg/kg	s-6 mg/kg	s-7 mg/kg	s-8 mg/kg	s-9 mg/kg	s-10 mg/kg	a-7 mg/kg
<16 mg/L	34 (39.5)	33 (38.4)	11 (12.8)	3 (3.5)	0	0	0	8 (15.1)
<20 mg/L	73 (84.9)	67 (77.9)	38 (44.2)	19 (22.1)	3 (3.5)	3 (3.5)	0	22 (41.5)
≥20 mg/L	13 (15.1)	19 (22.1)	48 (55.8)	67 (77.9)	83 (96.5)	83 (96.5)	86 (100)	31 (58.5)

a-5: actual dose given 5mg/kg; s5 –s10mg/kg: simulated doses using the formula: ‘peak concentration simulated dose = (peak concentration/actual dose) × simulated dose’; a-7: actual dose given 7mg/kg.

### Phase 2: Simulations

To determine which dose was needed to reach an adequate gentamicin peak concentration of ≥16mg/L, doses were simulated with a range of 5–10 mg/kg (Table 2). The simulations showed that with a dose of 7mg/kg 96.5% of patients would reach the target of ≥16mg/L, and 77.9% would reach a peak concentration of ≥20mg/L (Fig 1 and Table 3).

**Table 3:** Monitoring of renal function after the administration of a gentamicin dose of 5 and 7mg/kg.

n (%) or median [IQR]	5 mg/kg n = 86	7 mg/kg n = 53	*P* value
Baseline creatinine (μmol/L)	84 [71–116]	84 [68–108]	0.39
Admission creatinine (μmol/L)	90 [76–155]	95 [67–117]	0.22
Creatinine T1 (μmol/L)	87 [72–136]	95 [78–126]	0.71
Creatinine T2 (μmol/L)	91 [76–155]	93 [70–158]	0.96
Creatinine T3 (μmol/L)	91 [91–169]	74 [64–136]	0.10
AKI on admission (relative to baseline)	16/84 (19.0)	8/52 (15.4)	
Risk	9/84 (10.7)	6/52 (11.5)	
Injury	4/84 (4.8)	2/52 (3.8)	
Failure	3/84 (3.6)	0	
AKI on T3 (relative to admission; e.g. after the administration of gentamicin)	1/74 (1.4)	2/49 (4.1)	
Risk	1/74 (1.4)	1/49 (2.0)	
Injury	0	1/49 (2.0)	
Failure	0	0	

Baseline creatinine: most recent creatinine value prior to admission; Admission creatinine: creatinine value on admission; T1: 1–2 days after admission; T2: 3–7 days after admission; T3: 8–14 days after admission; AKI: acute kidney injury according to the RIFLE criteria [19].

### Phase 3: Confirmation of new gentamicin dosing regimen of 7mg/kg with peak concentrations

After the simulations, we assumed that a dose of 7mg/kg was needed to achieve an adequate gentamicin peak concentration in the majority of patients. A new dosing regimen was implemented in our hospital according to which a dose of 7mg/kg was administered to patients with sepsis at the ED. Gentamicin peak concentrations were measured according to protocol for confirmation of the simulated doses.

A total of 53 patients was included in this third phase. Of these patients, 66.0% was male with an age of 72 years [60–80] (Table 1). Median body weight was 78kg [67–89] and BMI was 26.5kg/mg^2^ [23.3–30.8]. No differences were found in baseline characteristics between the group receiving 5mg/kg, and the group receiving 7mg/kg. Patients were treated with a dose of 6.9 mg/kg [6.3–7.1] and reached a peak concentration of 21.8mg/L [18.0–26.0]. With a dose of 7mg/kg, a total of 15.1% of patients did not reach a peak concentration of ≥16mg/L, and 41.5% did not reach a peak concentration of ≥20mg/L, respectively. These findings are shown in Fig 1, with a similar pattern for the simulated dose of 7mg/kg, and the measured dose of 7mg/kg (*R* = 0.993, *P*<0.001).

### Renal function

All patients received a single dose of gentamicin as part of the empirical antibiotic treatment of sepsis. Creatinine values at baseline and at admission were similar in the group receiving 5mg/kg, and the group receiving 7mg/kg (84μmol/L vs. 84μmol/L, *P* = 0.39) (Table 3). After the administration of gentamicin, there were no differences between both groups regarding creatinine values during follow up of 14 days. The overall incidence of AKI relative to creatinine value at admission was low in both groups, with 1 patient (1.2%) with Risk in the 5mg/kg group, and 1 patient (4.2%) with Risk, and another one with Injury in the 7mg/kg group. No patients needed renal replacement therapy.

### Microbiological analysis

Of the 80 positive blood cultures with Gram negative bacteria taken at the ED in 2015, 67.5% were *E*. *coli*, 17.5% were *Klebsiella spp*., 11.2% were other Enterobacteriaceae, and 3.8% were *P*. *aeruginosa*. Of the Enterobacteriaceae, 58 strains (75.2%) had an MIC <2mg/L, and seven strains (9.1%) had an MIC = 2mg/L. Twelve (15.6%) had an MIC ≥4mg/L, and were resistant for gentamicin (Table 4).

**Table 4:** MIC distributions of positive blood cultures with Gram negative bacteria.

Micro-organism	Number (n%)	Gentamicin MIC distribution (n %)		
		MIC < 2 mg/L	MIC = 2 mg/L	MIC ≥ 4 mg/L
*E*. *coli*	54 (67.5)	41 (51.3)	6 (7.5)	7 (8.8)
*Klebsiella spp*.	14 (17.5)	11 (13.8)	0	3 (3.8)
Other Enterobacteriaceae	9 (11.2)	6 (7.5)	1 (1.3)	2 (2.5)
*P*. *aeruginosa*	3 (3.8)	2 (2.5)	1 (1.3)	0
Total	80 (100)	60 (75)	8 (10)	12 (15)

MIC: minimal inhibitory concentration (mg/L)

## Discussion

Our study shows that in patients presenting with sepsis at the ED a gentamicin dose of 7mg/kg is superior to 5mg/kg in order to achieve sufficient gentamicin peak concentrations of ≥16mg/L. This is in line with previous studies in ICU patients [8, 9].

Since insufficient gentamicin peak concentrations (i.e. <16mg/L) were measured in 39.5% of patients with sepsis at the ED after a dose of 5mg/kg, peak concentrations for higher doses of gentamicin were determined by simulation. According to these simulations, with a dose of 7mg/kg almost 95% of patients would reach a peak concentration of ≥16mg/L, which would be the preferred peak concentration for bacteria with an MIC of 2mg/L [7]. Nearly 10% of positive blood cultures with Enterobacteriaceae in our ED had an MIC of 2mg/L, which shows the need for adequate dosage of gentamicin during the empirical treatment of sepsis at the ED. This applies especially to countries with a higher prevalence of resistant micro-organisms compared to the Netherlands.

After the implementation of the new gentamicin dosing regimen, the measured peak concentrations corresponded well with our simulations. A gentamicin dose of 7mg/kg turned out to be sufficient in the majority (84.9%) of patients at the ED for Enterobacteriaceae with an MIC of 2mg/L.

There are concerns whether a short course of gentamicin in a dose of 5mg/kg leads to AKI and some question whether aminoglycosides are needed at all in the empirical treatment of sepsis [20]. These concerns will probably increase with the use of higher doses of gentamicin.

Although our study was not designed and powered to study the incidence of AKI, we did not find differences in the incidence of AKI after the administration of a single dose of gentamicin between the two groups (5 vs. 7mg/kg) and the overall incidence of AKI was very low. This is in line with Nicolau *et al*., where only a low incidence of nephrotoxicity (1.2%) was found after a short course of 7mg/kg of gentamicin in 2184 clinical patients (non-ICU and non-ED) [4]. In addition, a single dose of gentamicin 5mg/kg as part of the empirical treatment in 179 patients with sepsis at the ED, was not associated with an increased incidence of AKI in another study [21]. Therefore, the empirical use of aminoglycosides in patients with sepsis appears to be safe and in our opinion the advantages of a higher peak concentration outweigh the potential renal side effects, although our study is underpowered and not designed for this purpose.

Furthermore, aminoglycosides can reduce the use of 3^rd^ and 4^th^ generation cephalosporins and carbapenems. For example, in a country with a low prevalence of multidrug resistant micro-organisms such as the Netherlands, the resistance rates of Enterobacteriaceae in clinical blood isolates for amoxicillin/clavulanic acid and 2^nd^ generation cephalosporin’s range from 7–24% [22]. In the ICU [22], and in countries with a higher prevalence of multidrug resistant micro-organisms these numbers are higher [23]. However, the resistance rates of Enterobacteriaceae in non-ICU clinical blood isolates in the Netherlands are as low as 1–3% for the combination of gentamicin and amoxicillin/clavulanic acid or a 2^nd^ generation cephalosporin [22].

Our study has some limitations. First, it is a single-centre study, and second, we had to rely on the correctness of the blood sampling protocol. However, our ED staff is well trained and adhered well to the peak sampling protocol. Second, our study was not powered to assess differences in the incidence of AKI; therefore, conclusions regarding the renal safety of the higher gentamicin dosing regimen cannot be drawn from this study. Furthermore, when using the target peak concentration of 16-20mg/L for Enterobacteriaceae, dosage is based on the assumption that all gentamicin susceptible Enterobacteriaceae could have an MIC = 2mg/L. In our study only 9.1% of Enterobacteriaceae had an MIC = 2mg/L, resulting in over-treating the majority of patients using this target. However, at the time of administering antibiotics in sepsis at the ED, the MIC of the causative pathogen is unknown. Therefore, this over-treatment is inevitable, since we assume the worst case scenario of an MIC = 2mg/L when decisions are being made at the ED.

In conclusion, a gentamicin dose of 5mg/kg leads to inadequate peak concentrations in a significant proportion of patients with sepsis at the ED. Since it is essential that aminoglycosides are adequately dosed in order to achieve a maximal bactericidal effect in patients with sepsis, we advise that other EDs using gentamicin as part of their empirical treatment of sepsis increase the dose of gentamicin to 7mg/kg.

## Supporting information

S1 Dataset Anonymized database plos one—English.sav. (SAV)
